# Supplementary material for: Learning curve and surgical outcome of robotic assisted colorectal surgery with ERAS program
Source: Sci Rep. 2022 Nov 29;12:20566. doi: 10.1038/s41598-022-24665-w (PMC9709162; doi:10.1038/s41598-022-24665-w)
Supplement: Supplementary file 2 — Supplementary Information 2. [file 41598_2022_24665_MOESM2_ESM.zip › RAL Raw data-Ñ[▒K/110/OP N_ñw╝╨░O▒Kñσ.pdf]

W52 / W52

臺中榮民總醫院
手術室護理記錄

[redacted]

基本資料

- 室別： 開刀房-17室，手術科別： CRS，手術類別： 常規，日期： 2021/09/09
- 進入時間： 0725 ； 麻醉開始時間： 0755 ； Time Out時間： 0920 ； 劃刀時間： 0922 ； 手術結束時間： 1420 送出時間： 1435 ；
- 麻醉方式： GE
  診斷： Malignant tumor of rectum, L/3,LEFT
  手術名稱： Robot Assisted Low Anterior Resection
  手術局部用藥： 2%Xylocaine jelly
  術前預防性抗生素：
    - 抗生素名稱一： Veterin 1GM IVD/由麻醉人員給予
    - 時間： 0920
    - 上次簽章: 吳婉寧

  ，追加預防性抗生素：
    - 抗生素名稱一： Veterin 1GM IVD由麻醉護理師給予
    - 時間： 1320
    - 上次簽章: 王明盈
- 手術醫師： 蔣鋒帆，陳瑢夫，楊曙榮，洪浚嚴
- 麻醉醫師： VS 齊光彪
-
    - 刷手護士A ： 劉雅汶，時間： 0710，刷手護士B ： 羅媗鈴，時間： 0710，接替刷手一： 劉雅汶/廖子晴，時間： 1400
    - 流動護士A ： 吳婉寧，時間： 0710，流動護士B ： 許芷瑄，時間： 0710，接替流動一： 王明盈，時間： 1200

皮膚狀況

A-V Shunt： 無
術前： 完整
　記錄者： 上次簽章: 吳婉寧
術後： 完整
　記錄者： 上次簽章: 王明盈

手術準備

手術臥式 單一手術臥式

-
    - 手術臥式： 截石臥式
    - 使用附件： 溫毯 40 ℃，矽膠枕頭
    - 皮膚準備： 10%Aq Betadine，Hibiscrub，2% Chlorhexidine
    - 電燒： 使用電燒: 電極片位置： 其它： 電極迴路墊，雙極電燒，記錄者： 上次簽章: 吳婉寧

止血帶T： 無

植入物

無

標本送檢

- 細菌培養 無
- 細胞學 無
- 冰凍切片 無
- 病理標本
  組織名稱一： distal cutend
  ,
  組織名稱二： IMA Root
  ,

組織名稱三： rectum

- 截肢 無

標本交付 標本： 1 袋 ， 標本： 0 盒 ， 標本： 2 瓶 ， 細胞學： 0 管 ， Culture Tube： 0 管 ， 被交付醫師： CR 陳璿夫 ，

- 記錄者： 上次簽章: 王明盈
- 時間： 1345

傷口術後敷料

傷口敷料： 傷口敷料： 無

- 請於圖上標示部位：

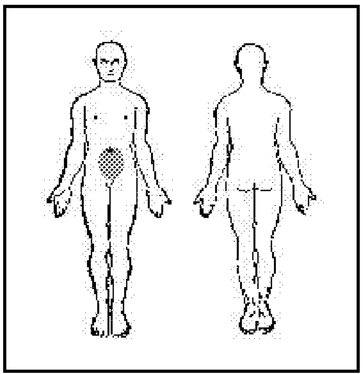

- 傷口敷料： 軟性敷料

導管

導管	天數	導管資訊	插入日期	插入時間	更換日期	拔除日期	拔除時間	拔除原因	預計拔除/更換	預計拔除/更換日期
Foley(1)	1	號碼:16 Fr.; 部位:膀胱;	2021/09/09	0830						
Sump NG Tube(1)	1	號碼:16 Fr.; 深度:Fix 60cm; 部位:胃;	2021/09/09	0910						
A-line (1)	1	號碼:20 G; 部位:左手;	2021/09/09	0820						
Double Lumen CVC(1)	1	號碼:8 fr; 深度:fix 15cm; 部位:subclavian (右); 象限:RU;	2021/09/09	0810						
Epidural PCA(1)	1	部位:硬脊膜外;	2021/09/09	0800						

用物計數

- 手術中出血量： minimal c.c.
- 手術中尿量： 750 c.c.
-
    - 其它： 無

用物計數： 多切口同時縫合

- 1.器械： 正確 ，2.縫針： 正確 ，3.刀片： 正確 ，4.紗布： 正確 ，5.腹部墊： 未使用 ，6.紗球： 未使用 ，7.PEANUTS ： 未使用 ，8.紗條： 未使用 ，9.絲帶： 未使用 ，10.針頭： 未使用 ，12.Boots： 未使用 ，13.Jelco： 未使用 ，14.棉條： 未使用 ，
    - 第一次：
        - 刷手護士 ： 劉雅汶/廖子晴
        - 流動護士： 上次簽章: 王明盈
        - 時間： 1330

,
    - 第二次：
        - 刷手護士 ： 劉雅汶/廖子晴
        - 流動護士： 上次簽章: 王明盈
        - 時間： 1345

,
    - 第三次：
        - 刷手護士 ： 劉雅汶/廖子晴

- 流動護士： 上次簽章: 王明盈
- 時間： 1410

備註

備註： 達文西30度鏡:2號

- 記錄者： 上次簽章: 吳婉寧
- 時間： 1043

填寫護理人員 王明盈

正式摘要

W52 / W52

doctorRank:B
